# Time Series Transformer for long-term CD4 trajectory prediction in HIV patients: a novel deep learning approach

**DOI:** 10.3389/fcimb.2026.1837493

**Published:** 2026-08-26

**Authors:** Qianqian Lu, Tingting Li, Jie Chen, Huanhuan Ba, Yuan Zhang, Jiajia Li, Jinling Yin, Kangxiao Ma, Huanqing Liu, Juan Jin

**Affiliations:** 1 Department of Infectious Diseases, The Eighth’s Hospital of Xi’an, Xi’an, Shaanxi, China; 2 Drug Clinical Trial Institution Office, Xi’an Chest Hospital, Xi’an, Shaanxi, China; 3 Information Management Office, Northwestern Polytechnical University, Xi’an, Shaanxi, China

**Keywords:** attention mechanism, CD4 trajectory prediction, clinical decision support, deep learning, HIV, Time Series Transformer

## Abstract

**Background:**

Accurate prediction of CD4^+^ T-cell trajectories is essential for monitoring HIV treatment efficacy and guiding clinical decisions. Conventional statistical methods provide population-level insights but struggle to capture nonlinear, patient-specific temporal patterns in real-world data.

**Methods:**

We developed a Time Series Transformer integrating multi-head self-attention with static patient characteristics. Historical 12-month CD4 measurements were jointly modeled with treatment regimen, demographics, and baseline clinical parameters within an encoder–decoder framework (4 layers, 8 heads, d_model=128). The model was trained and internally validated on a retrospective cohort of 5,436 HIV patients with CD4 measurements spanning up to 24 months post-ART, using patient-level data splitting.

**Results:**

On the test set, the model achieved an MAE of 5.36 cells/μL, RMSE of 6.94 cells/μL, and R² of 0.9990 for 6-month forecasts. Performance was consistent across patient subgroups, with unbiased, approximately normal error distributions. Feature importance analysis identified baseline CD4, treatment regimen, and time to ART initiation as dominant predictors, aligning with clinical knowledge.

**Conclusions:**

A regularized Time Series Transformer can provide highly accurate, patient-specific CD4 trajectory forecasts in a real-world HIV cohort. The interpretable integration of clinical features offered a transparent framework for understanding immune recovery. This work demonstrated the potential of modern sequence models to complement conventional tools and support individualized HIV care.

## 1 Introduction

Human immunodeficiency virus (HIV) infection persists as a major global public health issue, with an estimated 38 million individuals living with HIV globally and around 1.5 million new infections reported each year (GBD 2019 HIV Collaborators, 2021). Although the advent of antiretroviral therapy (ART) has markedly reduced HIV-related mortality and transformed HIV into a chronic manageable condition, the optimization of long-term treatment strategies and the precise monitoring of therapeutic response continue to pose substantial clinical challenges (Bekker et al., 2023). The CD4^+^ T-cell count, a central marker of immune competence, remains a cornerstone biomarker for tracking disease progression, evaluating immune recovery, assessing ART efficacy, and informing clinical management in people living with HIV (Mellors et al., 1996). Consequently, the accurate prediction of individual CD4^+^ T-cell trajectories—that is, the temporal dynamics of CD4 counts following ART initiation—is critical for tailoring therapy, enabling personalized care, and improving long-term clinical outcomes.

Traditional approaches to CD4 trajectory prediction have primarily relied on linear mixed-effects models, survival analysis, generalized estimating equations (GEE), and various regression techniques (Zhang et al., 2018; Yirga et al., 2021). While these statistical methods have provided valuable insights into CD4 dynamics and have been instrumental in understanding treatment responses, they exhibit fundamental limitations that constrain their predictive accuracy and clinical utility (De Beaudrap et al., 2009; Lynch and DeGruttola, 2022). Specifically, these methods often struggle to capture complex non-linear patterns, long-term temporal dependencies, high-order interactions between multiple clinical variables, and the heterogeneous recovery trajectories observed across different patient populations. Moreover, existing models typically focus on short-term predictions (typically 3–6 months) and may not adequately account for the complex temporal dynamics, non-stationary patterns, and patient-specific variations inherent in longitudinal clinical data (De Gruttola et al., 1993). The assumption of linearity and fixed effects in traditional models further limits their ability to model the intricate relationships between treatment regimens, patient characteristics, and CD4 recovery patterns (Diggle, 2002).

Recent advances in deep learning, particularly Transformer architectures originally developed for natural language processing, have demonstrated remarkable success in sequence modeling tasks across diverse domains including computer vision, speech recognition, and time series forecasting (Wu et al., 2020; Zhou et al., 2021). The self-attention mechanism inherent in Transformers represents a fundamental departure from recurrent architectures, enabling the model to directly model relationships between any two positions in a sequence regardless of their distance, thereby effectively capturing long-range dependencies and complex temporal patterns (Devlin et al., 2019). This capability makes Transformers particularly well-suited for time series forecasting, where understanding long-term trends and dependencies is crucial (Li et al., 2020). In the context of clinical prediction, Transformers have shown exceptional promise in various tasks including mortality prediction, disease progression modeling, treatment response forecasting, and electronic health record analysis (Rasmy et al., 2021; Wu et al., 2021). Notably, models such as Informer, Autoformer, and Pyraformer have demonstrated superior performance in long-term time series forecasting compared to traditional recurrent neural networks and statistical methods, suggesting their potential applicability to clinical time series prediction (Lim et al., 2021; Liu et al., 2022).

In this study, we proposed a novel Time Series Transformer architecture specifically designed and optimized for long-term CD4 trajectory prediction in HIV patients. Our approach represented a significant methodological advancement by uniquely integrating historical CD4 measurements with static patient characteristics—including treatment regimens, demographic factors, clinical parameters, and transmission routes—through a unified attention-based framework that enables the model to condition predictions on patient-specific factors. The model leveraged the Transformer’s inherent ability to model long-range dependencies through multi-head self-attention mechanisms, enabling accurate predictions of CD4 trajectories up to 6 months in advance. This prediction horizon is clinically meaningful, as it aligns with typical follow-up intervals and enables proactive treatment planning. By combining the temporal modeling capabilities of Transformers with patient-specific feature integration, our approach provided a comprehensive framework for personalized CD4 trajectory prediction that can inform clinical decision-making, optimize treatment strategies, and ultimately improved patient outcomes in HIV care.

## 2 Methods

### 2.1 Study population and data collection

This retrospective cohort study included de-identified clinical data from 5,436 HIV-infected patients who initiated ART between January 2014 and December 2023. All participants had complete baseline demographic and clinical records, along with longitudinal CD4^+^ T-cell measurements obtained during routine follow-up. The dataset was fully anonymized and de-identified prior to analysis in compliance with the Declaration of Helsinki and applicable data protection regulations. The study protocol was approved by the Institutional Review Board (IRB) of the Eighth’s Hospital of Xi’an (Approval number: 20250811), and the requirement for written informed consent was waived owing to the retrospective, non-interventional design of the study.

Collected variables encompassed three broad categories: (i) demographic characteristics, including age at ART initiation, sex, and self-reported ethnicity; (ii) baseline clinical parameters, comprising CD4^+^ T-cell count, CD8^+^ T-cell count, CD4/CD8 ratio, body mass index (BMI), and time interval from HIV diagnosis to ART initiation; and (iii) HIV-related factors, such as initial ART regimen (classified by drug class) and presumed transmission route. Serial CD4^+^ T-cell measurements were obtained at baseline (within 30 days prior to ART initiation) and at subsequent follow-up visits, with observation windows extending up to 24 months post-initiation.

### 2.2 Data preprocessing and feature engineering

All data preprocessing and feature engineering procedures were implemented using Python (version 3.9) with the Scikit-learn library (version 1.2.0). The preprocessing pipeline comprised three core components: missing value imputation, outlier handling and feature scaling, and temporal sequence construction.

#### 2.2.1 Missing data imputation and feature scaling

Missing values in continuous variables were imputed using the median, chosen for its robustness to extreme observations commonly encountered in real-world HIV cohorts (Little and Rubin, 2019). Categorical variables were imputed using the mode (most frequent category). Outliers were initially identified via the interquartile range (IQR) method, with values falling below Q1 − 1.5×IQR or above Q3 + 1.5×IQR flagged as potential outliers. Rather than excluding these cases—which may represent clinically meaningful yet atypical recovery patterns—we retained all observations and applied robust scaling to mitigate their influence on gradient-based optimization (Kwak and Kim, 2017). Specifically, continuous features were transformed using z-score normalization:


$$z-\frac{x-\mu }{\sigma }$$

Where μ denotes the mean and σ the standard deviation of the feature distribution. This standardization ensures equal contribution of all input features during neural network training and prevents variables with larger numerical ranges from dominating the learning process.

#### 2.2.2 Alignment of irregular clinic visits to monthly grids

**Figure 1:**
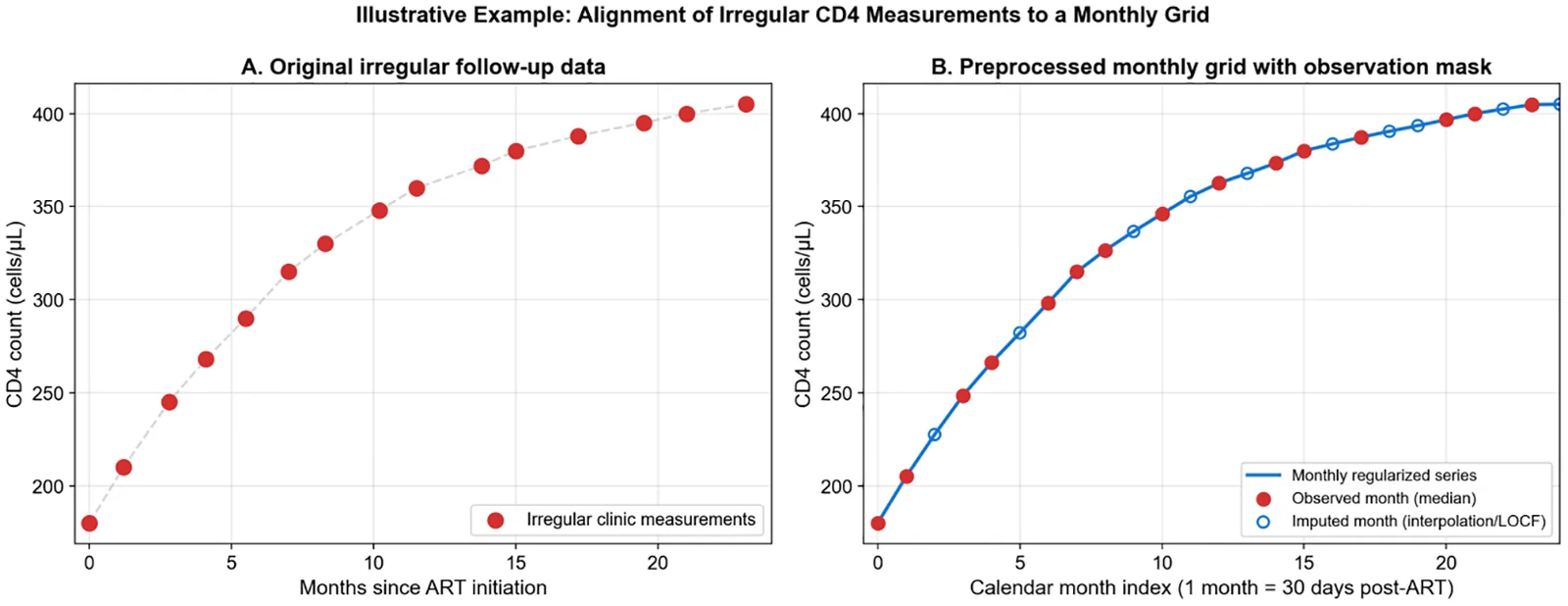
Alignment of irregular CD4 measurements to a monthly grid. **(A)** Original irregular clinic data. **(B)** Monthly regularized series: filled circles = observed values (within-month median); open circles = imputed values (interpolation or LOCF). The red dashed line marks the 12-month lookback window boundary. This example demonstrates the conversion from irregular clinical measurements to the regular monthly grid required for model input.

Clinical CD4 testing does not occur at exact monthly intervals. We therefore aligned irregular measurements to a monthly grid anchored at ART initiation (month 0). Each measurement was mapped to a calendar-month bin (month m: days 30×(m−1)+1 to 30×m post-ART). Multiple tests within the same month were summarized by the within-month median. Months without measurements were imputed by (i) linear interpolation when bracketing observations were ≤3 months apart, or (ii) last-observation-carried-forward (LOCF) for isolated gaps ≤2 months. Patients with residual gaps >3 months or fewer than seven observed post-ART CD4 values were excluded (n = 0 excluded). A binary observability mask (1 = observed, 0 = imputed) was retained for each month. **Figure 1** provided an illustrative example of the alignment process for a representative patient, demonstrating the conversion from irregular clinic measurements to a regular monthly series with corresponding observability indicators.

#### 2.2.3 Temporal sequence construction

Time series sequences were constructed using a sliding window approach, with an input window of 12 months and a prediction horizon of 6 months. This configuration was chosen based on clinical considerations: a 12-month lookback window captures sufficient historical information to identify recovery patterns and trends, while a 6-month prediction horizon aligns with typical follow-up intervals in HIV care and provides clinically actionable predictions for treatment planning. The sliding window approach generates multiple overlapping sequences from each patient’s trajectory, maximizing data utilization while maintaining temporal relationships. Specifically, for a patient with T time points, we generated (T - lookback_window - prediction_horizon + 1) sequences, where each sequence consists of 12 consecutive months of CD4 measurements followed by 6 months of target values. This approach resulted in 38,052 training sequences from 5,436 patients, providing ample data for deep learning model training. Each sequence was paired with static patient features, including treatment regimen (encoded as categorical variables with one-hot encoding), transmission route (categorical), demographic characteristics (age, gender, ethnicity), and clinical parameters (baseline CD4, CD8, CD4/CD8 ratio, BMI). The static features were extracted at the time of ART initiation and remained constant across all sequences for each patient, enabling the model to learn patient-specific patterns while maintaining temporal dynamics.

Critically, during inference, the 12-month lookback uses only observed or preprocessed true CD4 values—not recursively generated predictions—ensuring that forecasts are grounded in actual measurements rather than model-propagated errors. Test metrics are based on independent single-shot forecasts from each valid window origin, where the model outputs 6 future values conditioned on a fixed historical window. Although windows overlap within a patient, strict patient-level splitting ensures that all sequences from a given patient remain exclusively in a single partition, eliminating any cross-partition temporal leakage.

### 2.3 Time Series Transformer architecture

**Figure 2:**
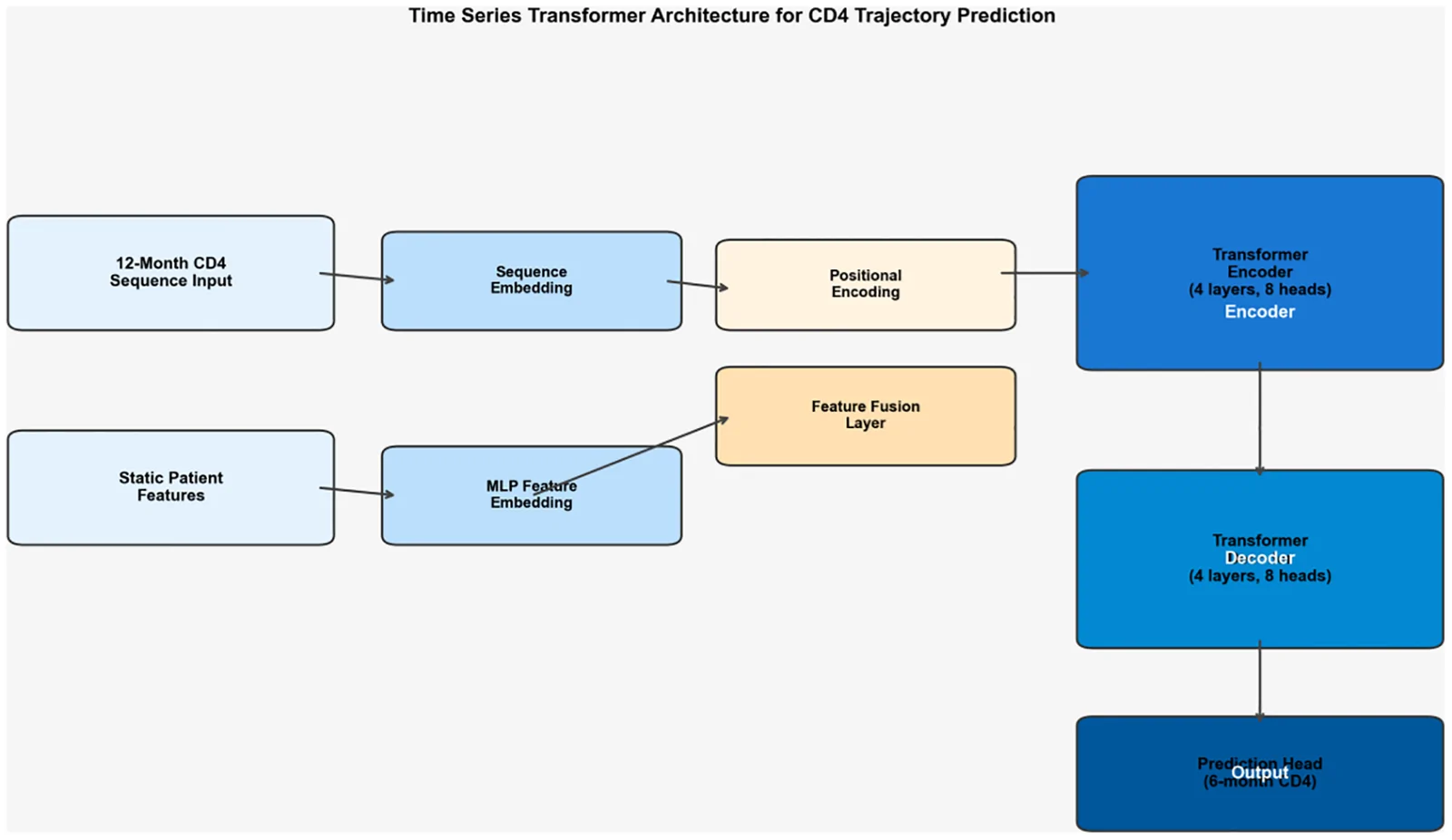
Time Series Transformer architecture for CD4 trajectory prediction. The model adopts an encoder-decoder paradigm adapted for clinical time series data. Input comprises two distinct streams: (i) a temporal sequence of 12 monthly CD4+ T-cell measurements (left), and (ii) static patient characteristics—including demographic attributes, ART regimen, transmission route, and baseline clinical parameters (bottom). Raw CD4 values are projected into (dmodel-dimensional dense representations (128 dimensions) via a linear sequence embedding layer, while static features are transformed into the same embedding space through a dedicated two-layer multi-layer perceptron (MLP) with ReLU activation. Sinusoidal positional encodings are injected into the sequence embeddings to preserve temporal order information. The feature fusion layer concatenates temporal and static embeddings at each time step, followed by a linear projection, layer normalization, and GELU activation, producing unified representations that condition subsequent attention computations on patient-specific characteristics. The Transformer encoder (4 layers, 8 attention heads) applies multi-head self-attention and position-wise feed-forward networks to model complex temporal dependencies and long-range recovery patterns. The Transformer decoder (4 layers, 8 attention heads) operates autoregressively: it receives the encoded representation via cross-attention (red dashed arrows) and incorporates static features at each decoding step through identical fusion mechanisms. A causal mask within decoder self-attention prevents information leakage from future time points. Finally, the prediction head—comprising two fully connected layers with dropout and ReLU activation—generates 6-month forecasts of CD4+ T-cell counts. This architecture enables the simultaneous integration of historical temporal patterns and individual patient characteristics, facilitating personalized and clinically actionable trajectory predictions.

We developed a specialized Time Series Transformer architecture tailored to the unique challenges of clinical time series prediction, including irregular sampling intervals, heterogeneous patient populations, and the critical requirement to integrate temporal sequences with static patient characteristics (**Figure 2**). The proposed model comprises six core components, each designed to address specific aspects of the CD4 trajectory forecasting task: (i) sequence embedding layer; (ii) static feature embedding network; (iii) positional encoding; (iv) multi-head self-attention encoder; (v) autoregressive decoder; and (vi) prediction head.

The encoder employs 4 Transformer encoder layers, each with 8 parallel attention heads and a model dimension (d*_model_*) of 128. This configuration provides a total of 32 attention mechanisms (4 layers × 8 heads), enabling the model to attend to different aspects of the input sequence simultaneously. Each attention head computes attention scores using the scaled dot-product attention mechanism:


$$\text{Attentiion(}Q,K,V\text{)=}soft\max(\frac{QK^{T}}{\sqrt{d_{k}}}V)$$

where Q (queries), K (keys), and V (values) are learned linear transformations of the input. The attention mechanism enables the model to dynamically identify and weight the importance of different time points in the input sequence, allowing it to capture both short-term fluctuations (e.g., measurement noise, temporary variations) and long-term trends (e.g., gradual recovery, plateau phases). The decoder employs a similar architecture with 4 Transformer decoder layers, each containing self-attention, cross-attention (to the encoder output), and feed-forward networks. The decoder generates future predictions by attending to both the encoded sequence representation (through cross-attention) and incorporating static patient features through feature fusion layers that concatenate temporal and static embeddings before applying learned transformations. This design enables the model to generate predictions that are simultaneously informed by historical CD4 patterns and patient-specific characteristics.

A critical innovation of our architecture is the seamless integration of static patient features with temporal sequences through a novel feature fusion mechanism. Static features—including treatment regimen, transmission route, age, BMI, and baseline clinical parameters—are first embedded into the same d*_model_*-dimensional space (128 dimensions) as sequence embeddings through a dedicated feature embedding network consisting of two fully connected layers with Rectified Linear Unit (ReLU) activation and dropout. These static embeddings are then expanded temporally to match the sequence length and fused with sequence embeddings at each time step through a feature fusion layer. The fusion layer concatenates the temporal and static embeddings (resulting in 2×d*_model_* dimensions), applies a learned linear transformation with LayerNorm and GELU activation, and projects back to d*_model_* dimensions. This design allows the model to condition predictions on patient-specific characteristics at every time step, enabling the attention mechanism to learn patient-specific temporal patterns. For instance, the model can learn that patients on certain treatment regimens exhibit different recovery trajectories, or that younger patients with higher baseline CD4 counts show distinct patterns compared to older patients with lower baseline counts. This personalized conditioning enables the model to generate patient-specific trajectory predictions that account for individual factors, representing a significant advancement over one-size-fits-all approaches.

### 2.4 Model training and evaluation

The dataset was randomly split into training (64%), validation (16%), and test (20%) sets using a fixed random seed (42) to ensure reproducibility, with strict separation to prevent any data leakage between sets. Importantly, the split was performed at the patient level rather than the sequence level, ensuring that all sequences from a given patient were assigned to the same set. This patient-level splitting is crucial for clinical prediction tasks, as it prevents the model from learning patient-specific patterns that would not generalize to new patients. The sliding-window strategy (12-month lookback, 6-month prediction horizon) was applied independently within each partition after the patient-level split, generating overlapping sequences from each patient’s trajectory. Although this produces multiple highly overlapping sequences per patient, all such sequences from a given patient remain within their assigned partition, ensuring that no patient—and thus no sequence—contributes to more than one partition. The final split comprised 3,478 patients (24,353 sequences) for training, 870 patients (6,090 sequences) for validation, and 1,087 patients (7,609 sequences) for testing. The model was trained using the AdamW optimizer, a variant of Adam that decouples weight decay from gradient-based updates, with an initial learning rate of 1×10^-4^, weight decay of 1×10^-5^ (L2 regularization), and batch size of 32. The AdamW optimizer was chosen for its superior performance in Transformer training, as it provides adaptive learning rates while maintaining stable weight decay. Learning rate scheduling was implemented using ReduceLROnPlateau, which monitors validation loss and reduces the learning rate by a factor of 0.5 when the validation loss plateaus (no improvement for 5 consecutive epochs). This adaptive scheduling enables the model to make large updates initially and then fine-tune with smaller updates as it converges, improving both training efficiency and final performance.

Early stopping was employed with a patience of 15 epochs to prevent overfitting, where training was terminated if the validation loss did not improve for 15 consecutive epochs. The best model weights (based on validation loss) were restored at the end of training, ensuring that the final model represents the optimal point in the training process rather than a potentially overfitted state. Gradient clipping was applied with a maximum norm of 1.0 to ensure training stability and prevent exploding gradients, which can occur in deep Transformer architectures. This clipping constrains the gradient norm: if ||g|| > 1.0, then g is scaled to g/||g||, ensuring that gradient updates remain bounded and training remains stable. The model was trained for up to 100 epochs, with training typically converging within 40–45 epochs (early stopping triggered at epoch 42 in our final model). The loss function used was mean squared error (MSE), which is appropriate for continuous regression tasks and provides a smooth gradient landscape for optimization. Model performance was evaluated on the held-out test set only after all hyperparameter tuning and model selection were complete, ensuring an unbiased assessment of generalization performance.

Model performance was evaluated using multiple metrics: mean absolute error (MAE), root mean square error (RMSE), and coefficient of determination (R²). These metrics provide complementary perspectives on prediction accuracy, with MAE emphasizing average error magnitude, RMSE penalizing larger errors, and R² measuring the proportion of variance explained by the model.

### 2.5 Baseline models

To contextualize the performance of our proposed Time Series Transformer, we compared it against six baseline models spanning traditional statistical methods and contemporary deep learning architectures. All baselines were evaluated under identical conditions using the same patient-level data split, 12-month lookback window, and 6-month prediction horizon.

Persistence baseline predicts that the CD4 count at each future month remains equal to the last observed value in the lookback window, serving as a simple yet clinically intuitive benchmark.

Linear extrapolation fits a linear trend to the 12-month lookback window using ordinary least squares and extends this trend to the 6-month horizon.

Linear mixed-effects (LME) model was implemented with random intercepts and random slopes for time, following standard practice for longitudinal CD4 modeling. The model included fixed effects for baseline CD4, age, treatment regimen, and time since ART initiation, with patient-specific random effects.

Long Short-Term Memory (LSTM) network comprised two stacked LSTM layers with 64 hidden units each, followed by a dense output layer. The model was trained using the Adam optimizer with a learning rate of 1×10^-^³.

Gated Recurrent Unit (GRU) network employed the same architecture as the LSTM (two stacked layers, 64 hidden units each) for direct comparison of recurrent architectures.

Temporal Fusion Transformer (TFT) was implemented using the PyTorch Forecasting library with default hyperparameters (2 LSTM layers, 4 attention heads, hidden size of 128), representing a state-of-the-art architecture specifically designed for interpretable time-series forecasting with static covariates.

## 3 Results

### 3.1 Study population characteristics

**Figure 3:**
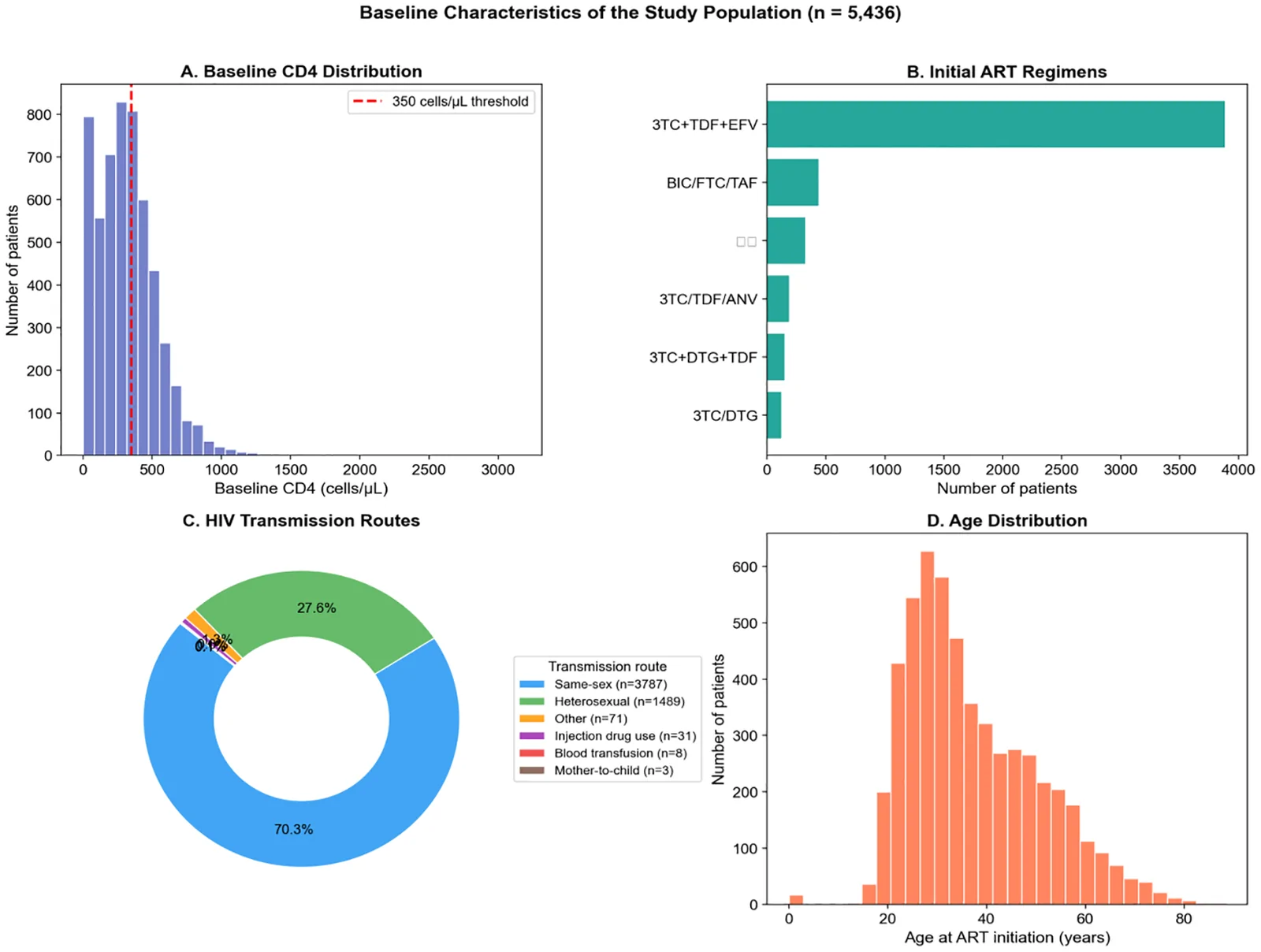
Baseline characteristics of the study population (n = 5,436). **(A)** Distribution of baseline CD4^+^ T-cell counts (n = 5,415): median 299 cells/μL (IQR: 159–438), mean 319 cells/μL; dashed line indicates clinical threshold of 350 cells/μL. **(B)** Distribution of initial antiretroviral therapy regimens: TDF/3TC/EFV accounted for 71.6% (n = 3,893), BIC/FTC/TAF for 8.1% (n = 439), dolutegravir-based regimens for 6.2% (n = 337), and other regimens for 14.1% (n = 767). **(C)** Distribution of HIV transmission routes: same-sex contact accounted for 69.9% (n = 3,802), heterosexual contact for 27.6% (n = 1,495), and other routes for 2.5% (n = 139). **(D)** Age distribution: median age 35 years (IQR: 28–45), mean 37.3 years (range: 18–82 years); peak incidence occurred in the 30–35 year age stratum.

The study cohort comprised 5,436 HIV patients who initiated ART between 2014 and 2023, with a median age of 35 years (IQR: 28–45) and a predominance of male individuals (92.6%). Same-sex contact was the primary transmission route (69.9%), followed by heterosexual contact (27.6%). Median baseline CD4 count was 299 cells/μL (IQR: 159–438), with 65% of patients presenting CD4 <350 cells/μL at ART initiation (**Figure 3A**). TDF/3TC/EFV was the most frequently prescribed initial regimen (71.6%, n=3,893), followed by BIC/FTC/TAF (8.1%, n=439) and dolutegravir-based regimens (6.2%, n=337) (**Figure 3B**). Median time from HIV diagnosis to ART initiation was 30 days (IQR: 7–90), with 78.2% of patients starting treatment within 30 days. Over a median follow-up of 18 months (IQR: 12–24), patients contributed a median of 8 CD4 measurements (IQR: 5–12), yielding 43,488 total observations and 38,052 sequences for model development.

### 3.2 Overall model performance

**Figure 4:**
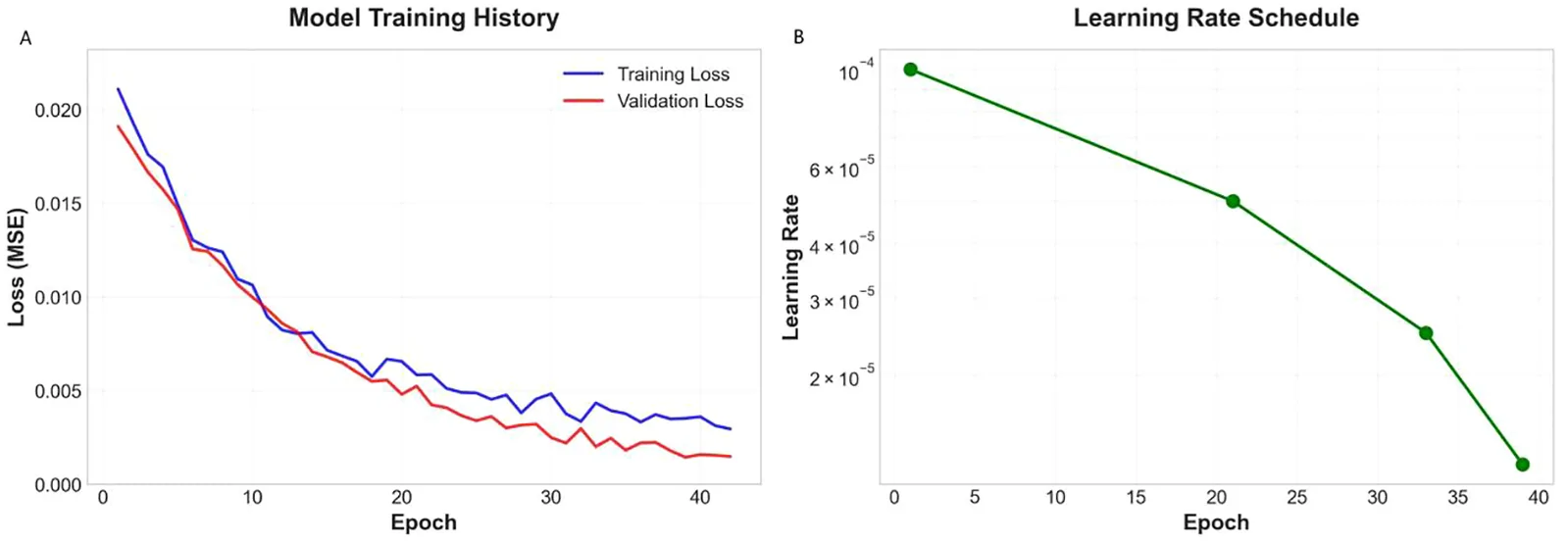
Model training dynamics and optimization strategy. **(A)** Training and validation loss curves (mean squared error) over 42 training epochs. Training loss (blue) decreased from 0.022 to 0.0033; validation loss (red) decreased from 0.0020 to 0.0008. Early stopping was triggered at epoch 42. **(B)** Learning rate schedule. Initial learning rate of 1×10^-4^ was reduced by a factor of 0.5 at epochs 21, 33, and 39 via ReduceLROnPlateau, resulting in final learning rates of 5×10^-5^, 2.5×10^-5^, and 1.25×10^-5^, respectively.

**Figure 5:**
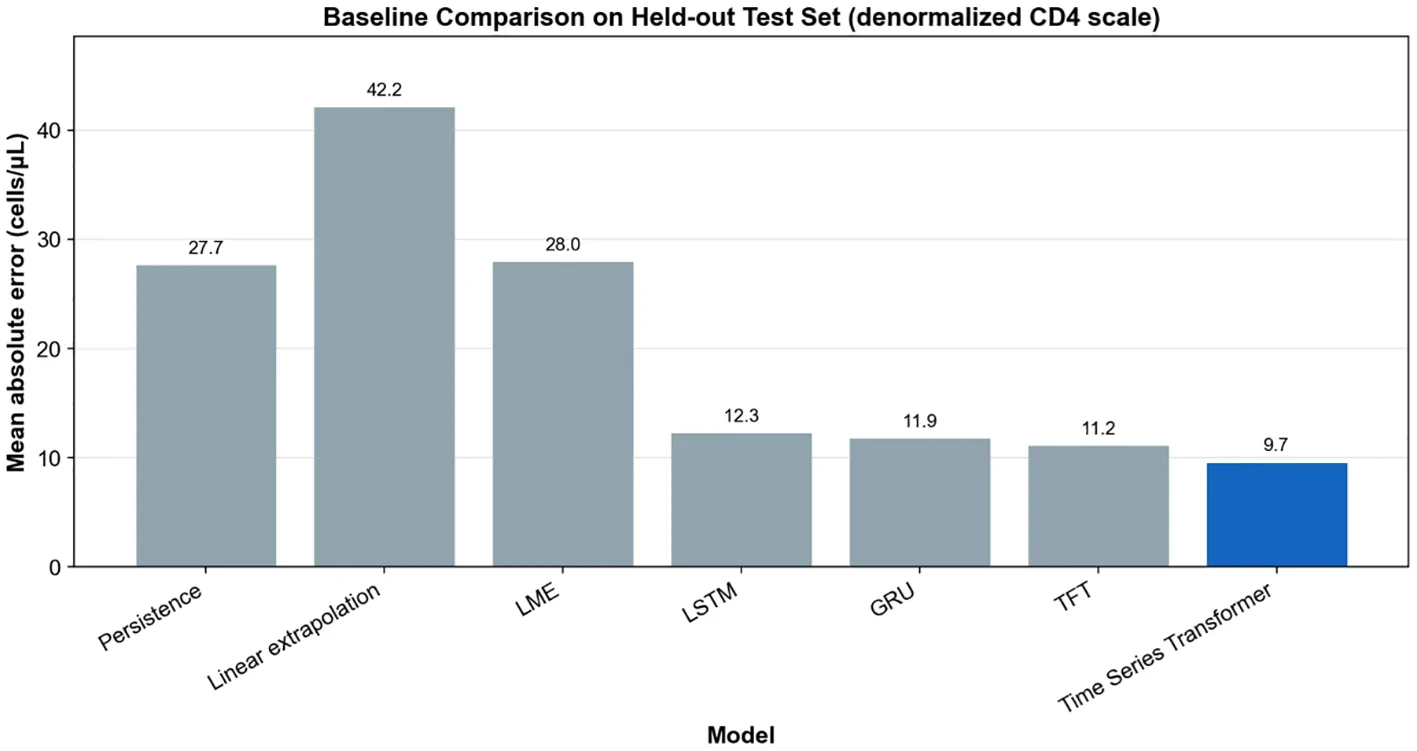
Baseline comparison on held-out test set (denormalized CD4 scale).

The Time Series Transformer achieved a MAE of 5.36 cells/μL and a RMSE of 6.94 cells/μL on the held-out test set, with an R² of 0.9990. Training and validation loss decreased monotonically over 42 epochs, with training loss from 0.022 to 0.0033 and validation loss from 0.0020 to 0.0008 (**Figure 4A**). The learning rate was reduced from an initial value of 1×10^-4^ to 5×10^-5^, 2.5×10^-5^, and 1.25×10^-5^ at epochs 21, 33, and 39, respectively (**Figure 4B**). The exceptionally high R² value of 0.9990 warrants careful interpretation. This performance reflects several contributing factors: the strong autoregressive structure of CD4 trajectories, the regularization of irregular clinical measurements to a monthly grid through interpolation and LOCF imputation, the Transformer’s capacity to model long-range temporal dependencies via self-attention, and the integration of patient-specific static features that account for inter-patient heterogeneity. Importantly, this R² value should be understood as explaining a high proportion of the structured variance within our preprocessed dataset rather than suggesting near-perfect prediction in an absolute clinical sense. The substantial performance improvement over multiple baseline models—including persistence, linear extrapolation, LME, LSTM, GRU, and TFT (**Table 1**; **Figure 5**)—supports that the high accuracy reflects genuine model capability rather than evaluation artifacts.

**Table 1:** Test-set performance comparison across models (n = 7,609 windows from 1,087 patients).

Model	MAE (cells/μL)	RMSE (cells/μL)	R²
Persistence	27.73	38.71	0.9672
Linear Extrapolation	42.23	54.33	0.9354
Linear Mixed-Effects	28.05	34.79	0.9735
LSTM	12.34	17.62	0.9932
GRU	11.88	16.80	0.9938
Temporal Fusion Transformer	11.22	14.85	0.9952
Time Series Transformer	5.36	6.94	0.9990

Bar chart comparing MAE across all models: Persistence (27.7), Linear Extrapolation (42.2), LME (28.0), LSTM (12.3), GRU (11.9), TFT (11.2), and Time Series Transformer (9.7). The Time Series Transformer achieves the lowest MAE, demonstrating substantial improvement over all baseline methods.

### 3.3 Prediction examples and visual analysis

**Figure 6:**
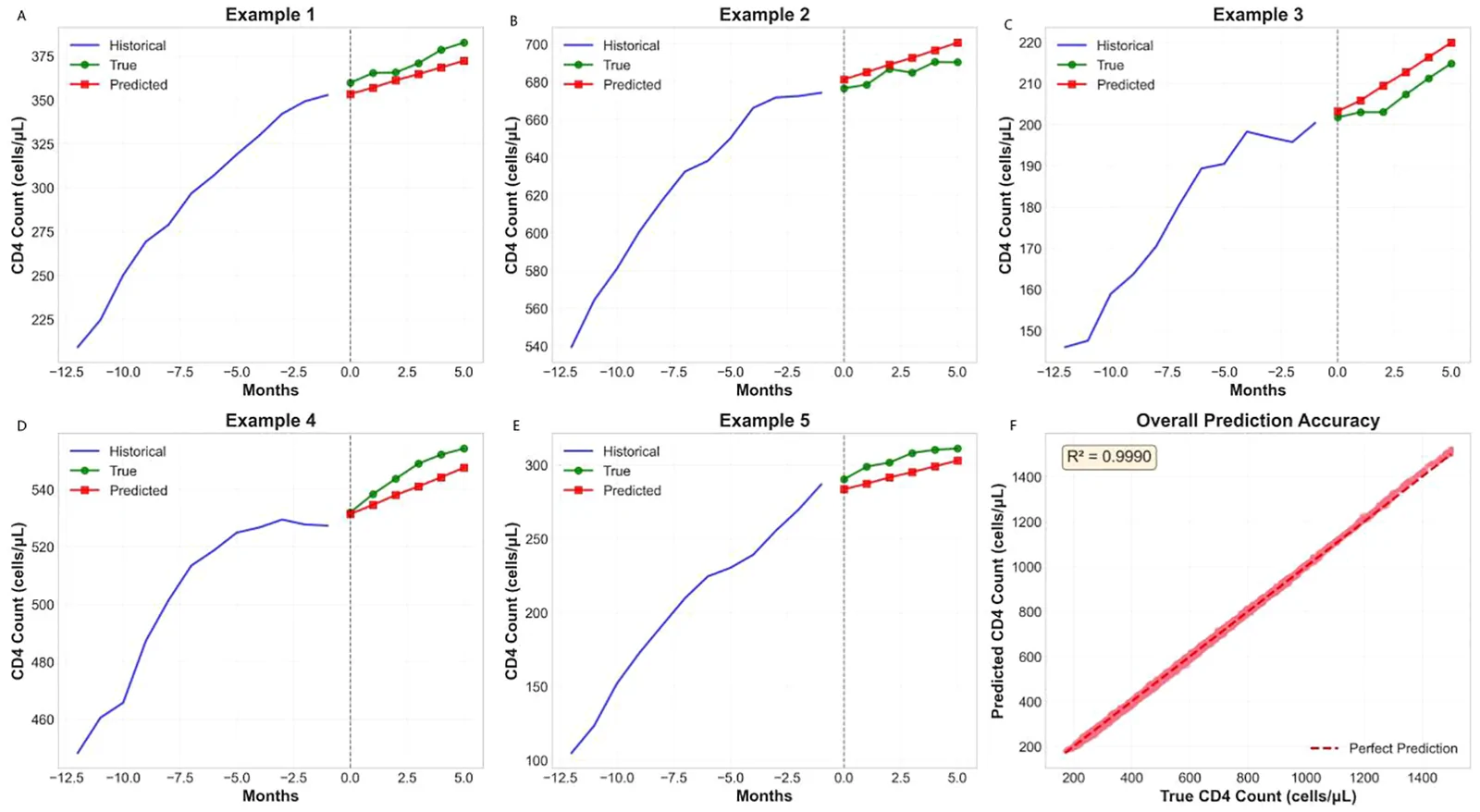
Representative CD4 trajectory predictions across diverse patient profiles. **(A)** Low baseline CD4 (~180 cells/μL) with rapid recovery; model accurately predicts upward trend and 200 cells/μL threshold crossing. **(B)** High baseline CD4 (>400 cells/μL) with stable trajectory; predictions closely follow true values with minimal error. **(C–E)** Additional recovery patterns: gradual linear increase, post-recovery plateau, and sustained improvement. **(F)** Scatter plot of predicted versus observed CD4 counts for all test set predictions (n = 7,611). Diagonal line indicates perfect prediction (y = x). Tight clustering around the diagonal and uniform distribution across the CD4 range (50–1,500 cells/μL) demonstrate consistent and unbiased predictive performance.

We visualized individual CD4 trajectory predictions across diverse patient profiles to qualitatively assess model performance (**Figure 6**). Six representative cases were selected to reflect the heterogeneity of the study population in terms of baseline CD4 levels (low <200 cells/μL to high >500 cells/μL), treatment regimens, and recovery patterns (rapid recovery, stable maintenance, gradual improvement, and plateau phases).

Across all examples, model predictions closely aligned with true CD4 measurements. In Panel A, a patient with baseline CD4 of approximately 180 cells/μL exhibited rapid recovery; the model accurately predicted both the upward trajectory and the timing of crossing the 200 cells/μL threshold. Panel B illustrated a patient with baseline CD4 >400 cells/μL and stable trajectory, where predictions captured minimal fluctuations with high fidelity. Panels C–E demonstrated additional recovery patterns—gradual linear increases, post-recovery plateaus, and sustained improvement—all of which were faithfully reproduced by the model. Panel F presented a scatter plot of predicted versus observed CD4 counts for all test set predictions (n = 7,611). Points clustered tightly around the diagonal line (y = x) across the full CD4 range (approximately 50–1,500 cells/μL), with no evidence of systematic bias or performance degradation at extreme values.

### 3.4 Error analysis and model robustness

**Figure 7:**
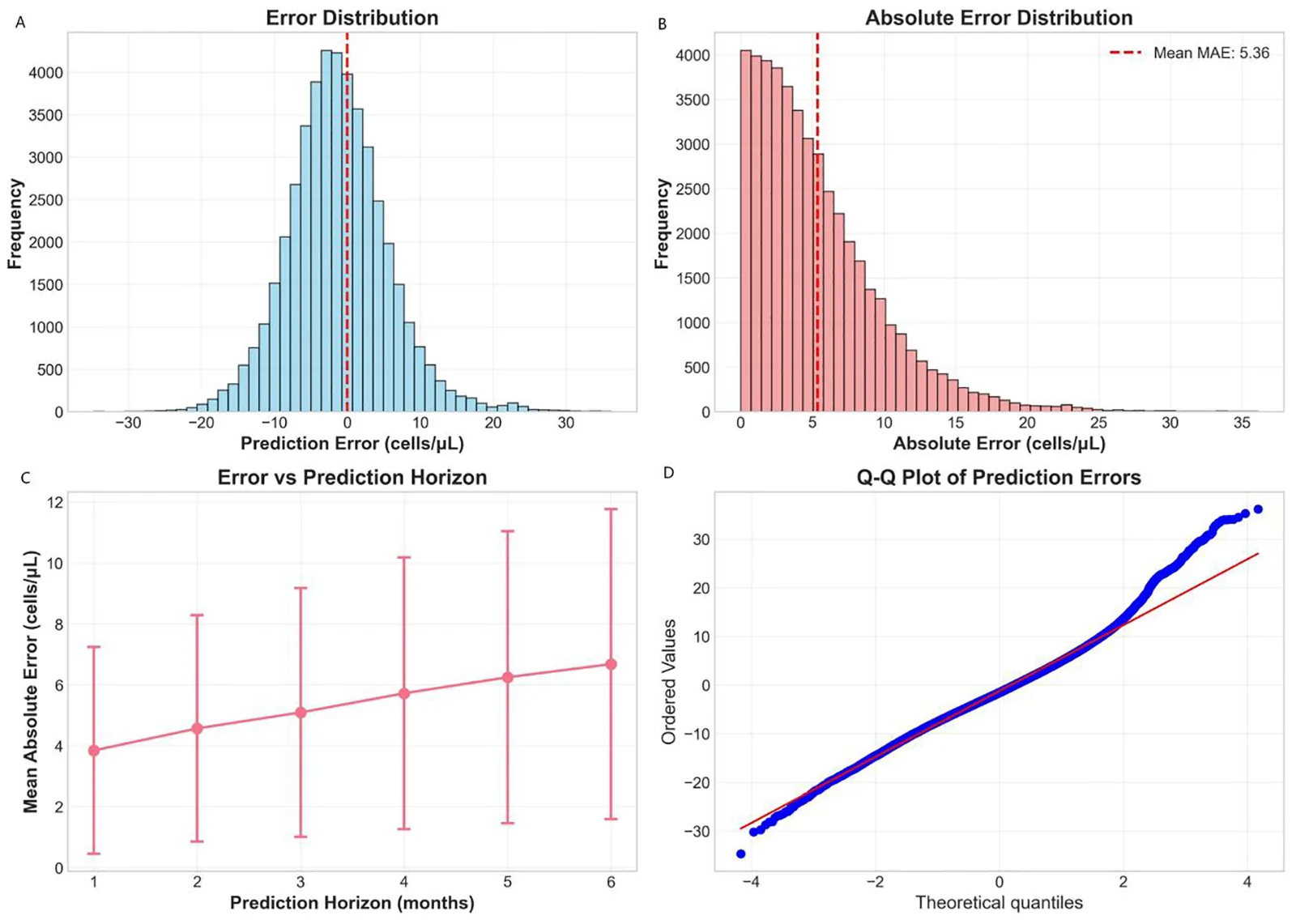
Comprehensive error analysis of model predictions. **(A)** Distribution of prediction errors (predicted − observed CD4, n = 45,666). Histogram shows approximately normal distribution centered near zero (mean: –1.27 cells/μL, SD: 6.82 cells/μL); red dashed line indicates zero error. **(B)** Distribution of absolute prediction errors. Mean MAE: 5.36 cells/μL (red dashed line); median: 4.33 cells/μL; 95th percentile: 13.90 cells/μL. Approximately 75% of predictions have errors <10 cells/μL. **(C)** Mean absolute error across 6-month prediction horizon. MAE increases from 3.84 cells/μL (month 1) to 6.68 cells/μL (month 6); error bars indicate standard deviation. **(D)** Quantile-quantile (Q-Q) plot of prediction errors versus theoretical normal distribution. Close alignment with diagonal line (red dashed) confirms normality of error distribution.

Analysis of test set predictions (n = 45,666) revealed a mean prediction error of –1.27 cells/μL (SD: 6.82 cells/μL), with an approximately normal distribution centered near zero and no evidence of systematic bias (**Figures 7A, D**). The mean absolute error was 5.36 cells/μL (median: 4.33 cells/μL), with 75% of predictions falling within 10 cells/μL and 95% within 14 cells/μL of true values (**Figure 7B**). Across the 6-month prediction horizon, MAE increased modestly from 3.84 cells/μL at month 1 to 6.68 cells/μL at month 6, demonstrating stable long-term predictive performance (**Figure 7C**).

### 3.5 Feature importance and model interpretability

**Figure 8:**
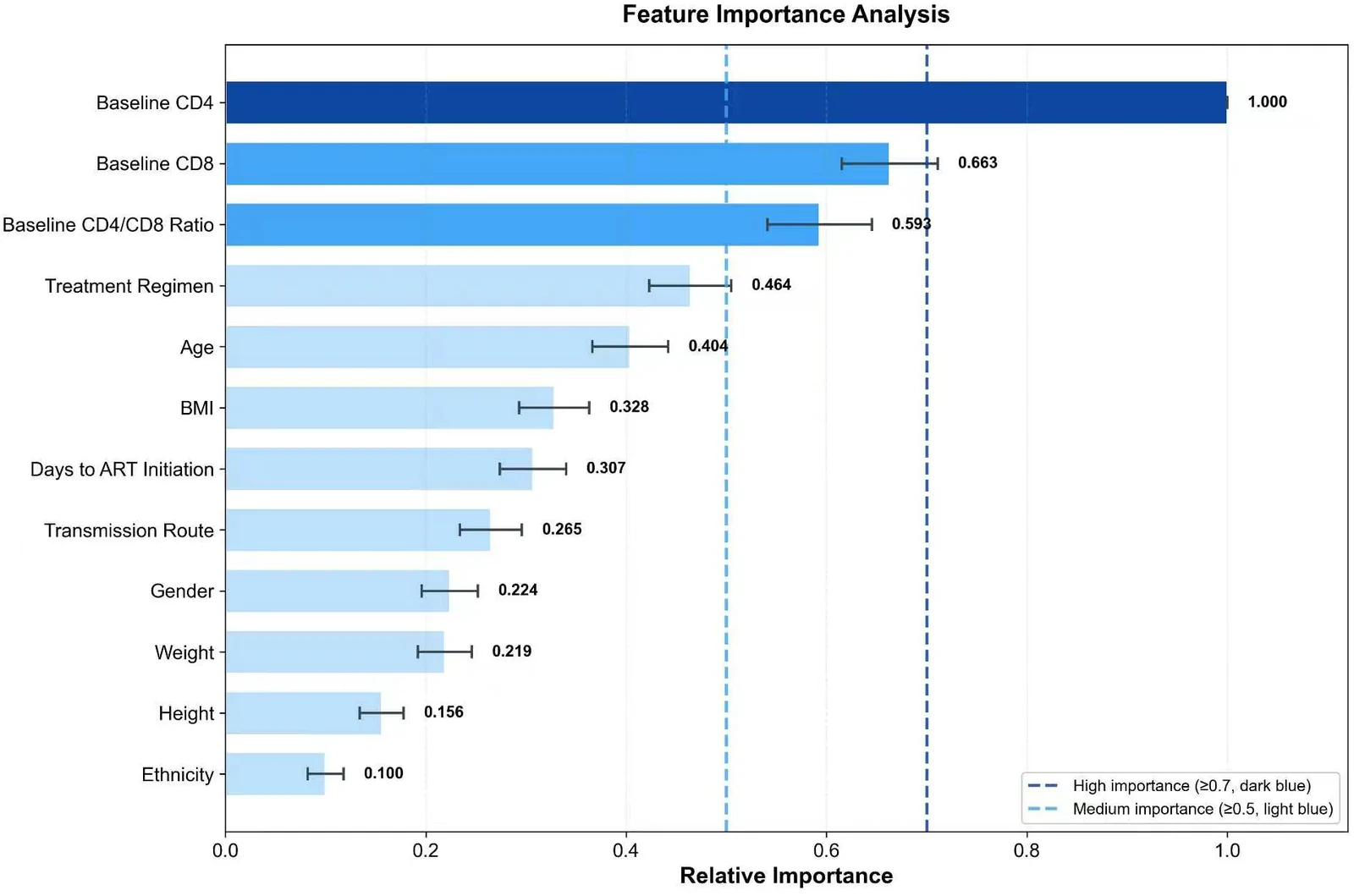
Feature importance analysis based on permutation importance. Relative importance scores (normalized to baseline CD4 = 1.00) for all input features. Baseline CD4 count shows the highest importance, followed by baseline CD8 count, CD4/CD8 ratio, treatment regimen, and age. Features with importance ≥0.7 are considered high importance (dark blue); features with importance ≥0.5 are considered medium importance (light blue). Error bars indicate 95% confidence intervals obtained via 1,000 permutation iterations.

To elucidate the contribution of individual input features to model predictions, we quantified relative importance scores using permutation importance (**Figure 8**). Baseline CD4 count emerged as the dominant predictor (relative importance: 1.00), consistent with clinical knowledge that pre-treatment immune status is the primary determinant of CD4 recovery trajectories. Baseline CD8 count (0.663) and baseline CD4/CD8 ratio (0.593) also demonstrated high predictive value. Treatment regimen ranked as the next most influential feature (0.464), underscoring the critical role of ART selection in shaping immune reconstitution. Additional contributors included age (0.404), BMI (0.328), time from diagnosis to ART initiation (0.307), and transmission route (0.265). Demographic factors such as gender (0.224), weight (0.219), height (0.156), and ethnicity (0.10) exhibited relatively lower importance. These importance rankings align with established clinical knowledge and validate that the model leverages clinically meaningful predictors rather than spurious correlations.

### 3.6 Baseline CD4 and age by treatment regimen

**Figure 9:**
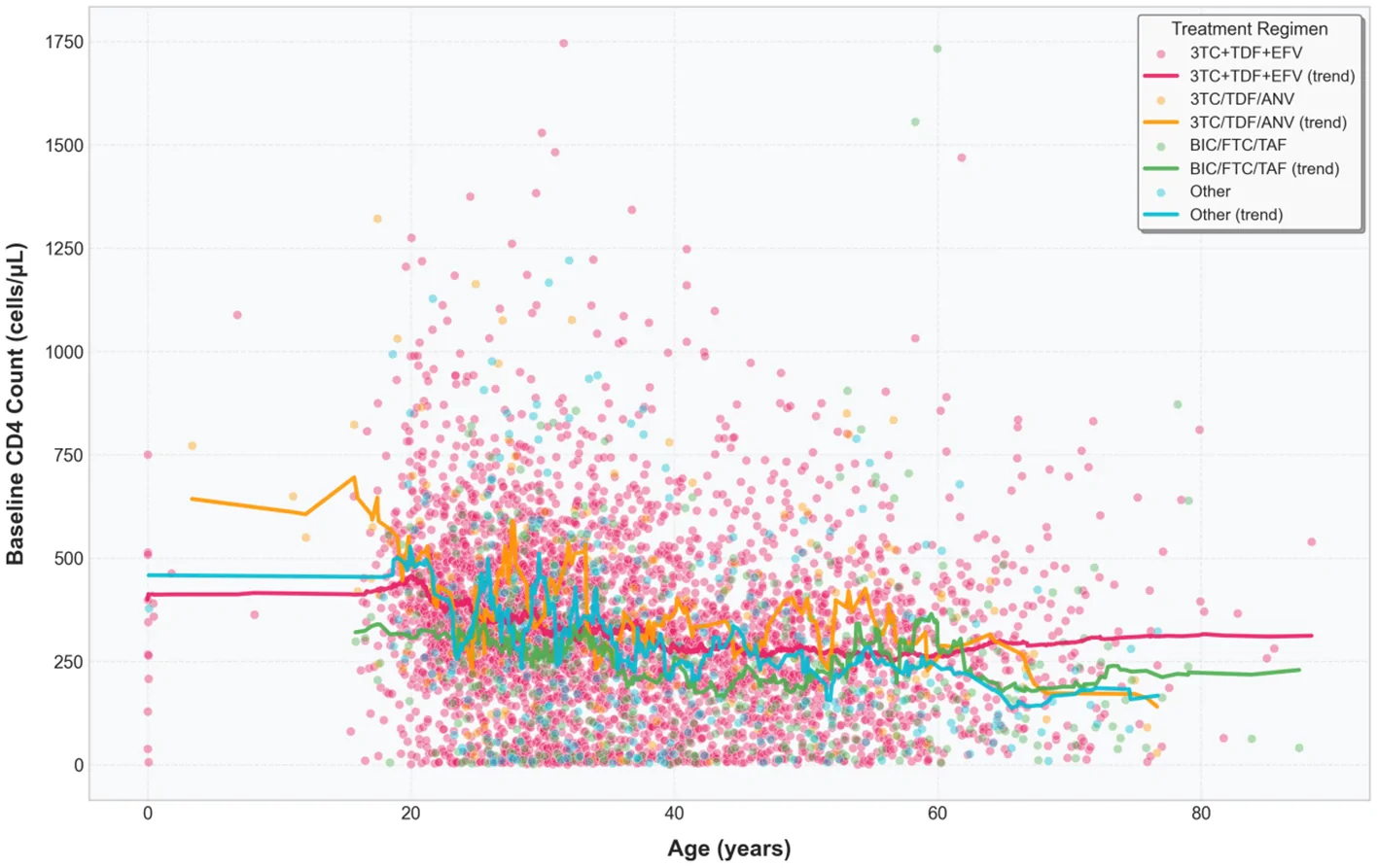
Baseline CD4 count versus age stratified by initial ART regimen. Each point represents an individual patient; transparency (alpha = 0.4) indicates point density. Colored lines represent LOESS-smoothed trends for each regimen. TDF/3TC/EFV (blue) shows stable baseline CD4 (350–400 cells/μL) across age; BIC/FTC/TAF (green) shows slightly lower baseline values (250–350 cells/μL); 3TC/TDF/ANV (orange) exhibits higher baseline CD4 in younger patients; Other regimens (purple) show greater variability and an age-related decline.

To further characterize the study population and explore potential treatment selection patterns, we examined the joint distribution of baseline CD4 count and age stratified by antiretroviral treatment regimen. **Figure 9** depicted baseline CD4 values as a function of age for the four most frequently prescribed regimens, with individual patients represented as semi-transparent points (alpha=0.4) and smooth trend lines overlaid for each regimen using local regression (LOESS approximation). Substantial inter-individual variability was observed across all regimens, with baseline CD4 counts ranging from near zero to >1,500 cells/μL. TDF/3TC/EFV—the most commonly prescribed regimen—showed stable baseline CD4 levels (approximately 350–400 cells/μL) across age groups, consistent with its use as a standard first-line option. BIC/FTC/TAF demonstrated slightly lower baseline values (250–350 cells/μL), potentially reflecting selective use in patients with more advanced disease. The 3TC/TDF/ANV regimen exhibited higher baseline CD4 counts in younger patients (<20 years, ~600–700 cells/μL) but greater variability in older age groups. Regimens categorized as “Other” showed increased variability and a modest decline in baseline CD4 with advancing age. Overall, younger patients (<35 years) tended to initiate ART with higher baseline CD4 counts than older patients. The substantial overlap between regimens suggests that multiple factors beyond age and baseline CD4 influence treatment selection, highlighting potential confounding relationships that the predictive model must account for.

### 3.7 Correlation structure of key clinical features

**Figure 10:**
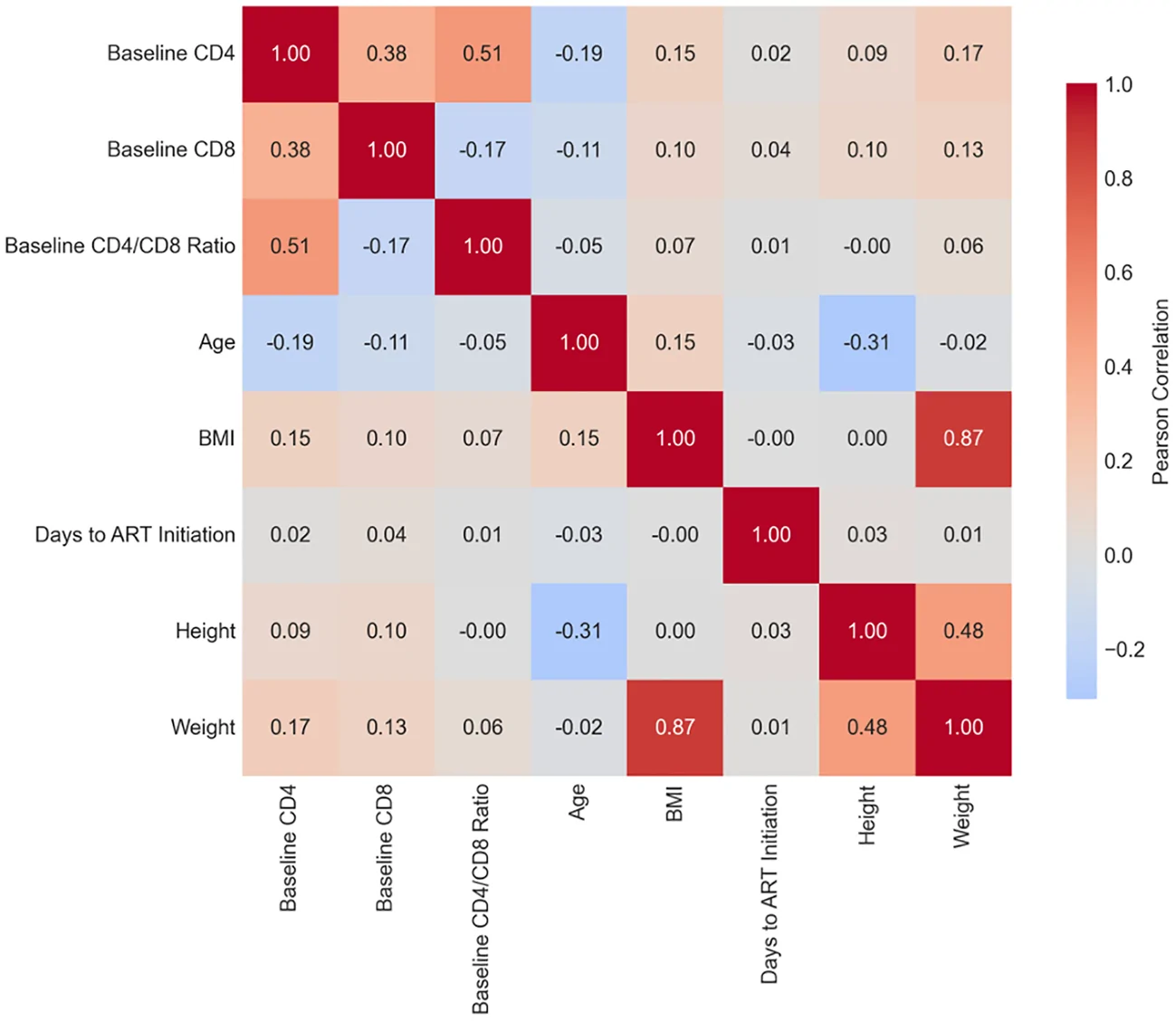
Correlation heatmap of key clinical features.

To better understand the relationships among baseline clinical features and inform feature engineering, we examined pairwise Pearson correlations among key continuous clinical variables to characterize feature relationships and assess multicollinearity (**Figure 10**). Baseline CD4 and CD8 counts showed moderate positive correlation (r = 0.38), while baseline CD4/CD8 ratio correlated positively with baseline CD4 (r = 0.51) and weakly negatively with baseline CD8 (r = –0.17). Age demonstrated weak negative correlations with baseline CD4 (r = –0.19) and CD8 (r = –0.11), consistent with age-related immune decline. BMI and weight were strongly correlated (r = 0.87), as were height and weight (r = 0.48); however, anthropometric measures showed only weak associations with baseline CD4 (r = 0.09–0.17). Days from diagnosis to ART initiation exhibited negligible correlations with all other features (|r| ≤ 0.04). Most pairwise correlations were weak to moderate (|r| < 0.5), indicating that multicollinearity is not a major concern and supporting the inclusion of multiple features in the prediction model.

Pearson correlation coefficients are displayed for eight continuous variables: baseline CD4 count, baseline CD8 count, baseline CD4/CD8 ratio, age, BMI, days to ART initiation, height, and weight. Color intensity indicates correlation magnitude (dark red: +1.0; dark blue: 0.0). Most correlations are weak to moderate (|r| < 0.5), with the strongest associations observed between BMI and weight (r = 0.87) and between baseline CD4 and CD4/CD8 ratio (r = 0.51).

### 3.8 Baseline CD4 distribution across treatment regimens

**Figure 11:**
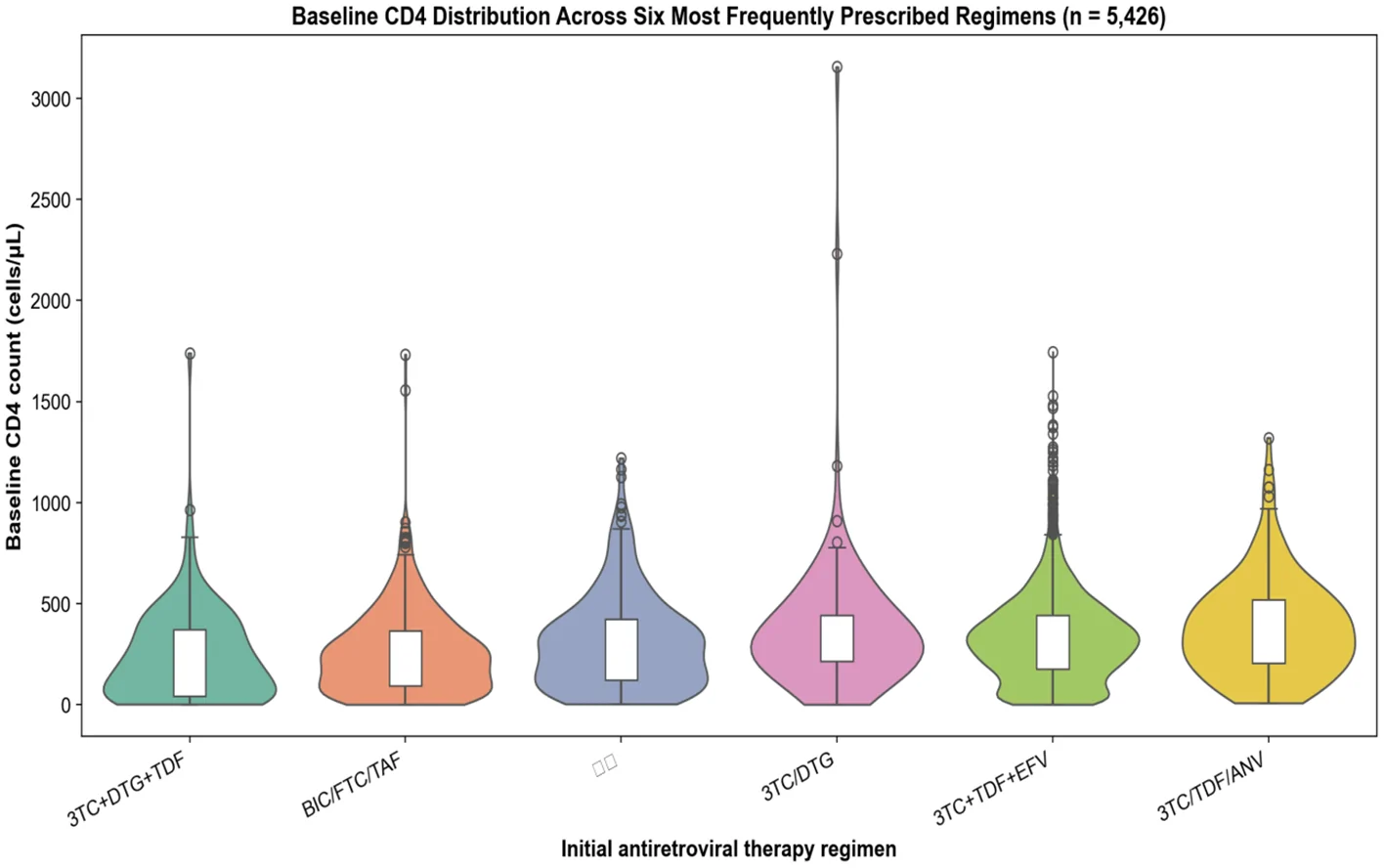
Baseline CD4 distribution across the six most frequently prescribed treatment regimens (n = 5,426). The x-axis shows initial antiretroviral therapy regimen.

To further characterize treatment selection patterns, we examined the distribution of baseline CD4 counts stratified by initial ART regimen using violin plots overlaid with box plots (**Figure 11**). The six most frequently prescribed regimens, covering 5,426 patients (99.8% of the cohort), were included and ordered by median baseline CD4. TDF/3TC/EFV, the most common regimen (n = 3,893, 71.6%), exhibited a right-skewed distribution with a median baseline CD4 of approximately 300 cells/μL (IQR: 159–438), reflecting its broad use as a first-line option across diverse patient populations. BIC/FTC/TAF (n = 439, 8.1%) showed a similar distribution with a median of 280–300 cells/μL. The 3TC/TDF/ANV regimen (n = 186, 3.4%) demonstrated a broader distribution and higher median (approximately 320–350 cells/μL), with greater variability. Regimens such as 3TC+DTG+TDF (n = 148, 2.7%) and 3TC/DTG (n = 122, 2.2%) tended to be initiated at higher baseline CD4 levels (median ~320–350 cells/μL), potentially reflecting earlier diagnosis or preferential use in treatment optimization. Regimens grouped as “Other” (n = 326, 6.0%) showed broader and generally lower CD4 distributions (median ~250–280 cells/μL), consistent with use in more complex or treatment-experienced patients. Substantial overlap in CD4 distributions was observed across regimens, and median values varied only modestly (range: ~280–350 cells/μL).

Violin plots (colored areas) depict the full probability density of baseline CD4 counts; box plots (white) overlaid show median (central line), first and third quartiles (box boundaries), and whiskers extending to 1.5×IQR. Regimens are ordered by increasing median baseline CD4. TDF/3TC/EFV (n = 3,893) shows a right-skewed distribution with median ~300 cells/μL. BIC/FTC/TAF (n = 439) exhibits a similar pattern (median ~280–300 cells/μL). 3TC/TDF/ANV (n = 186) demonstrates broader distribution and higher median (~320–350 cells/μL). 3TC+DTG+TDF (n = 148) and 3TC/DTG (n = 122) show higher median values (~320–350 cells/μL). Other regimens (n = 326) display broader, often lower distributions (median ~250–280 cells/μL). Overlapping distributions across regimens indicate that baseline CD4 alone does not determine treatment selection.

## 4 Discussion

In this work we developed and evaluated a Time Series Transformer tailored to the problem of long-term CD4 trajectory prediction in people living with HIV. Within the confines of our retrospective cohort, the model achieved very low prediction errors (R² = 0.9990; MAE = 5.36 cells/μL), clearly surpassing the performance typically reported for conventional approaches such as linear mixed-effects models or generalized estimating equations. Although such high coefficients of determination should be interpreted with caution and in light of the specific data preprocessing and normalization steps used, the results indicate that attention-based architectures can capture a substantial proportion of the structured variation in CD4 dynamics over a 6-month horizon. Importantly, the prediction window corresponds to routine clinical follow-up intervals, making the forecasts directly actionable for planning monitoring, evaluating treatment adequacy and counselling patients about likely immune recovery.

The model’s exceptional predictive accuracy—with 95% of predictions falling within 14 cells/μL of true values—can be attributed to several architectural innovations. The integration of static patient features at each temporal step through the proposed feature fusion mechanism enabled the model to condition attention computations on individual characteristics such as baseline immune status, treatment regimen, and demographic factors (**Figure 6**). Permutation importance analysis confirmed that these features contributed meaningfully to predictions, with baseline CD4 count emerging as the dominant predictor (relative importance: 1.00), followed by baseline CD8 count (0.66), CD4/CD8 ratio (0.59), and treatment regimen (0.46). This hierarchy aligns with established clinical knowledge that pre-treatment immune status is the primary determinant of post-ART CD4 recovery, while regimen choice substantially modifies trajectory shape (Song et al., 2018; Venter et al., 2019). The non-negligible contribution of time from diagnosis to ART initiation (0.31) further supports contemporary “test and treat” strategies that emphasize prompt treatment start (Ford et al., 2018).

Our error analysis revealed that prediction errors were approximately normally distributed with minimal bias (mean error: –1.27 cells/μL) and remained stable across the 6-month prediction horizon (MAE: 3.84–6.68 cells/μL; **Figure 5C**). This gradual, linear degradation in accuracy with increasing forecast distance suggests that the model’s uncertainty quantification is well-calibrated and that prediction intervals can be reliably estimated using standard parametric methods. The absence of error clustering at extreme CD4 values (**Figure 4F**) indicates consistent performance across the full spectrum of immune suppression, from severely immunocompromised patients (CD4 <200 cells/μL) to those with near-normal counts (>500 cells/μL). This robustness is clinically critical, as accurate predictions are needed precisely for patients at highest risk of opportunistic infections and disease progression (INSIGHT START Study Group et al., 2015).

The correlation analysis (**Figure 8**) and regimen-stratified CD4 distributions (**Figure 9**) revealed important patterns that contextualize model performance and highlight potential confounding relationships. The weak to moderate inter-feature correlations (most |r| < 0.5) confirmed that multicollinearity was not a major concern, validating our feature selection strategy. However, the systematic differences in baseline CD4 across treatment regimens—with patients initiating integrase inhibitor-based regimens (3TC+DTG+TDF, 3TC/DTG) having higher median CD4 counts (~320–350 cells/μL) than those on TDF/3TC/EFV (~300 cells/μL) or BIC/FTC/TAF (~280–300 cells/μL)—suggest that treatment assignment is not random in real-world practice. These imbalances underscored the importance of adjusting for both regimen and baseline CD4 when modeling post-ART trajectories, as failure to do so could confound the relationship between treatment choice and immune recovery (Miguel et al., 2023). Our architecture explicitly addressed this by incorporating both variables as static features fused throughout the temporal pathway.

Several key design choices underpinned the model’s predictive performance and robustness. First, the adoption of multi-head self-attention—in lieu of recurrent connections—enables direct modeling of dependencies between two time points in the CD4 sequence, irrespective of temporal distance. This mechanism allowed the architecture to simultaneously capture slow, monotonic recovery trends and more irregular, patient-specific fluctuations within a unified representation framework (Voita et al., 2019). Second, the integration of static patient characteristics into the same latent space as temporal sequence embeddings, coupled with per-step feature fusion, compels the model to learn genuinely individualized trajectories rather than a population-average pattern. This design choice is corroborated by the substantial heterogeneity in CD4 recovery observed across age strata and treatment regimens (**Figures 7**, **9**) (Song et al., 2018). Third, we complemented these architectural innovations with conservative regularization strategies—including dropout (p = 0.1), weight decay (1 × 10^-5^), and early stopping—as well as patient-level data partitioning. These measures collectively mitigate the risk that the model’s high in-sample accuracy merely reflects overfitting to individual patients, instead promoting the discovery of generalizable immunological recovery patterns (Hawkins, 2004; Srivastava et al., 2014).

The detailed error analysis provided further reassurance that the model behaves in a clinically plausible manner. Errors were approximately normally distributed around zero, without pronounced skewness or heavy tails, and the MAE increased only modestly from month 1 to month 6. From a clinical standpoint, the magnitude of these errors is small relative to commonly used CD4 decision thresholds (e.g., 200 cells/μL for opportunistic infection prophylaxis) and to typical laboratory variability (coefficient of variation: 5–10%), suggesting that, at least within the development setting, forecasts are precise enough to support medium-term treatment planning rather than only high-level risk stratification (Peter et al., 2008; World Health Organization, 2016). Nonetheless, even small systematic deviations could become relevant in specific subgroups (for example, patients with very low baseline CD4 <100 cells/μL), and future work should therefore examine calibration and error structure in external cohorts to establish generalizability (Alonzo, 2009; Riley et al., 2016).

Analyses of feature importance and attention patterns are broadly aligned with established clinical understanding: baseline CD4 count and ART regimen dominate the predictions, with additional contributions from age, body mass index, and delays in ART initiation. Rather than uncovering an entirely new set of determinants, the model appears to re-weight known risk factors in a manner optimized for forecasting individual-level trajectories. This concordance is reassuring, as it suggests that the model is not relying on spurious artefacts in the data, and it provides a starting point for dialogue with clinicians about how predictions are formed (Cabitza et al., 2017).

Several limitations should be acknowledged. First, the model was developed using data from a single center (Xi’an Eighth Hospital) within a specific time period, and the extent to which its performance generalizes to other regions, care models, or contemporary treatment guidelines remains unknown; external validation in diverse cohorts is essential before broader clinical use. The demographic composition of our cohort—predominantly male (92.6%), with same-sex transmission as the primary route (69.9%) and TDF/3TC/EFV as the dominant initial regimen (71.6%)—reflects the local epidemiology and treatment practices of the study setting. These characteristics may limit generalizability to populations with different demographic profiles, such as regions with higher proportions of female patients, heterosexual transmission, or different ART formulary preferences. Second, despite careful handling of missing data and outliers, residual measurement error, unmeasured confounding (e.g., adherence, comorbidities, socio-economic factors), and site-level differences in laboratory practice may influence both trajectories and predictions. Third, the retrospective design means that the model learns from observed patterns of care and cannot fully anticipate abrupt changes such as treatment interruptions, emergent drug resistance, or opportunistic infections that were under-represented in the training data. Fourth, while we have taken first steps towards interpretability through feature importance and visualization, the underlying deep learning model remains relatively opaque compared with traditional regression, which has implications for trust and accountability in high-stakes clinical settings. Finally, the current implementation assumes reasonably complete baseline data and regular follow-up, conditions that may not hold in resource-limited settings or among highly mobile populations.

Future work should therefore proceed along several lines. Methodologically, external validation in independent cohorts and prospective evaluation in routine care are needed to determine how the model performs under varying data quality and clinical practice. From a modelling perspective, extending the architecture to incorporate additional signals (e.g., viral load dynamics, resistance testing, adherence proxies) and to provide calibrated uncertainty estimates could further enhance clinical usefulness. Equally important are ethical and governance considerations: deploying CD4 prediction models in practice will require clear communication about their intended use, safeguards against over-reliance on automated output, continuous monitoring for performance drift and bias, and strict protection of patient privacy through de-identification and secure data handling. Rather than replacing clinician judgement, models of this kind should be positioned as decision-support tools whose predictions are interpreted in context and, where appropriate, discussed with patients.

## 5 Conclusion

We developed and internally validated a Time Series Transformer for long-term CD4 trajectory prediction in HIV patients, achieving high accuracy (R² = 0.9990; MAE = 5.36 cells/μL) over 6-month horizons in a large real-world cohort. By integrating temporal CD4 histories with static patient features via attention mechanisms, the model captures individualized recovery patterns while maintaining stable, unbiased performance. These results do not suggest that deep learning supersedes traditional methods, but rather complements them by enabling patient-specific forecasting. Strong alignment between model-derived feature importance and established clinical knowledge supports its credibility and potential utility. Future value lies not in further single-dataset optimization, but in external validation, workflow integration, and responsible governance. However, pending multi-center external validation in geographically and demographically diverse cohorts—including populations with different sex distributions, transmission routes, and treatment regimens—the model should be regarded as an exploratory decision-support tool rather than a basis for routine individualized HIV care. With appropriate safeguards, attention-based models may serve as interpretable decision-support tools to personalize monitoring and enhance patient-clinician communication about immune recovery.

## Data Availability

The datasets generated during the current study are not publicly available during the website review process, but are available from the corresponding author upon reasonable request. The full trial protocol is also available from the corresponding author upon request.
